# Action and Emotion Recognition from Point Light Displays: An Investigation of Gender Differences

**DOI:** 10.1371/journal.pone.0020989

**Published:** 2011-06-09

**Authors:** Kaat Alaerts, Evelien Nackaerts, Pieter Meyns, Stephan P. Swinnen, Nicole Wenderoth

**Affiliations:** Motor Control Laboratory, Research Centre of Movement Control and Neuroplasticity, Department of Biomedical Kinesiology, Group Biomedical Sciences, Katholieke Universiteit Leuven, Heverlee, Belgium; Cuban Neuroscience Center, Cuba

## Abstract

Folk psychology advocates the existence of gender differences in socio-cognitive functions such as ‘reading’ the mental states of others or discerning subtle differences in body-language. A female advantage has been demonstrated for emotion recognition from facial expressions, but virtually nothing is known about gender differences in recognizing bodily stimuli or body language. The aim of the present study was to investigate potential gender differences in a series of tasks, involving the recognition of distinct features from point light displays (PLDs) depicting bodily movements of a male and female actor. Although recognition scores were considerably high at the overall group level, female participants were more accurate than males in recognizing the depicted *actions* from PLDs. Response times were significantly higher for males compared to females on PLD recognition tasks involving (i) the general recognition of *‘biological motion’* versus ‘non-biological’ (or ‘scrambled’ motion); or (ii) the recognition of the *‘emotional state’* of the PLD-figures. No gender differences were revealed for a control test (involving the identification of a color change in one of the dots) and for recognizing the gender of the PLD-figure. In addition, previous findings of a female advantage on a facial emotion recognition test (the ‘Reading the Mind in the Eyes Test’ (Baron-Cohen, 2001)) were replicated in this study. Interestingly, a strong correlation was revealed between emotion recognition from *bodily* PLDs versus *facial* cues. This relationship indicates that inter-individual or gender-dependent differences in recognizing emotions are relatively generalized across *facial* and *bodily* emotion perception. Moreover, the tight correlation between a subject's ability to discern subtle emotional cues from PLDs and the subject's ability to basically discriminate biological from non-biological motion provides indications that differences in emotion recognition may - at least to some degree – be related to more basic differences in processing biological motion per se.

## Introduction

In everyday life, humans are constantly observing and interpreting the movements of others in an attempt to deduce their moods and emotional states. Folk psychology advocates the existence of gender differences in a number of these socio-cognitive functions such as ‘reading’ the mental states of others or discerning subtle differences in others body language. However, from an empirical perspective, the issue of gender differences in recognizing emotions is still a topic of debate. Studies using self-report questionnaires have revealed that females have higher empathy scores than males [1]–[4]. However, it is possible that the answers of participants might have been associated with indices of social desirability, potentially leading to biased results [3], [5]. Next to self-reports, the majority of studies addressing gender differences in the socio-cognitive domain employed paradigms involving emotion recognition from *facial* expressions. A meta-analysis on this topic revealed that 80% of studies show a female advantage [6], [7], however with relatively small effect sizes. Other studies even showed no gender differences in the recognition of facially expressed emotions [8], [9]. Recently it has been suggested that this inconsistency across studies can be explained by differences in the nature of stimuli, such that studies using emotional expressions of high intensity show fewer differences between male and female decoders than those using subtle expressions with less intensity [10].

However, facial expressions are not the only source for conveying emotionally relevant information. In every-day situations, other sources - such as the communicator's body language or “*bodily kinematics*” - might be important as well, especially when facial expressions are inconsistent or unavailable to the observer.

Concerning this topic, neuroscience and social cognition research are increasingly focusing on the role of the observer's own motor system in understanding or ‘reading’ others bodily kinematics [11]–[14]. Already within the framework of the social-cognitive simulation theory [15], [16] and the ideomotor theory [17], it was posited that the ‘understanding of other's actions and behavior’ may be essentially motor, rather than sensory in nature. The ideomotor principle - as first contended by James (1890) - assumed that “every representation of a movement awakens in some degree the actual movement which is its object” [18] and that common perceptual-motor representations are formed by the correlated experience of executing and perceiving actions. Accordingly, simulating other's actions by matching perceived movements onto the observers' own motor system has been proposed to be the general mechanism by which observers can read or understand the actions, intentions or emotions of others [11]–[14].

First neurophysiological evidence for the ‘embodied simulation theory’ was provided by the discovery of mirror neurons in the macaque monkey brain. Using single neuron recordings, the group of Rizzolatti et al., identified the existence of a particular class of neurons in the ventral premotor [19] and later in parietal cortices [20], which fire when the monkey performs a specific action, and also when it observes the same action performed by another individual. First indications of the existence of a mirror neuron system (MNS) in the human brain emerged from transcranial magnetic stimulation (TMS) studies showing ‘resonating’ activity in the observer's motor system when movements of others are observed [21]–[23], and similar findings on resonant activation of the motor cortex during mere observation of actions were provided from studies using electroencephalography (EEG) [24], [25] and magneto-encephalo-graphy (MEG) [26]. To date, also a large number of brain imaging studies have explored which brain regions become increasingly activated during the observation of other's actions and both the inferior frontal gyrus (IFG) and the inferior parietal lobule (IPL) have consistently been identified to be key areas of the human mirror system [27]–[29]. Based on its remarkable properties, the human MNS is hypothesized to be the neural mechanism by which observed movements are simulated or matched onto the observer's own motor system in order to read or understand the actions, intentions or emotions of others (i.e., embodied simulation) [13], [30]. Interestingly, several neurophysiological studies addressed the issue of gender differences in the MNS, and all reported mirror activations to be generally stronger in female compared to male participants [31]–[33]. Also voxel-based morphometry studies demonstrated that female participants display larger gray matter volumes than male participants in regions of the mirror system [34], [35]. To date, however, the issue of *behavioral* gender differences in action or emotion understanding remains fairly unexplored.

Prompted by findings of *neurophysiological* gender differences in mirror system functioning, the present study aimed to investigate whether gender differences are also quantifiable on *behavioral* tasks involving the understanding or reading of other's bodily kinematics. In our paradigm, point light displays (PLD) were used, in which biological motions are depicted solely by the kinematics of light points placed on the joints of an actor [36]. We employed these highly simplified versions of biological motion to exclude that the results were influenced by differences in stimulus intensity as reported for complex and more natural stimuli such as facial expressions [10]. Although PLDs lack visual properties such as color, shading, and contours, they are easily recognized as depicting biological motion [37] and have repeatedly been shown to activate the human MNS [38]–[41]. Using fMRI, Saygin et al. (2004) reported point-light biological motion animations to yield activity in frontal areas of the action observation network [38]. Similar patterns of results were found using EEG during the perception of PLDs depicting bodily actions [41], intentions or emotions [40]. In both studies, mu rhythm suppression – indicative of resonant mirror activity - was found during the perception of point-light induced biological motion but not during observation of non-biological motion displays. Also recent data from a lesion study showed that the ability to recognize PL biological motion directly relies upon and requires neuronal resources that are part of the action observation or mirror neuron system (MNS) [39].

Biological motion as depicted by PLDs, has been shown to contain all sorts of socially relevant information about human agents such as the kind of action they are executing [42], their gender [43], [44], their intentions and even their emotional state [45], [46]. In a series of experiments, we explored whether gender differences exist for the recognition of several ‘social’ features from PLDs. In a first experiment, we explored potential gender differences in the recognition of some basic aspects, such as (i) the ***‘displayed actions’*** or (ii) the point light figures' ***‘gender’***. In a second experiment, recognition of more subtle socially relevant cues, such as the ***‘emotional state’*** of the displayed point light figures was explored. An additional aim of the second experiment was to investigate whether potential gender differences in discerning socially relevant information from PLDs pertained to tasks involving the basic discrimination of ***‘biological motion’*** from ‘non-biological’ motion. In this task, subjects were required to indicate whether or not they recognized ‘a person’ in a series of human PLDs (‘biological motion’) or phase scrambled versions of the same PLD (‘non-biological motion’).

Based on the previously described stronger mirror system activation in females compared to males, we hypothesized the existence of behavioral gender differences (better performance in females) for (i) discerning subtle cues on the *emotional state* of the point light figure, and (potentially to a lesser extent) for (ii) recognizing the *displayed actions* and (ii) *gender* of the PLDs. Moreover, we additionally hypothesized that gender differences would pertain to the basic discrimination of ‘biological’ from ‘non-biological’ motion. No group differences were expected for a control PLD perception task, involving the identification of color changes in the moving point-light dots.

In experiment 2, we additionally tested whether the ability to recognize emotions from *bodily* PLD kinematics is correlated to the ability to recognize emotions from *facial* cues, as assessed by the ‘Reading the Mind in the Eyes Test’ (revised version) [47]. This test was previously developed as a measure of adult ‘mentalizing’ and has been shown to be a standardized and sensitive test to reveal subtle individual differences in emotion recognition from the eye region of different faces.

## Materials and Methods

### Ethics Statement

Written informed consent was obtained from all participants prior to the experiment. Consent forms and study design were approved by the local Ethics Committee for Biomedical Research at the Katholieke Universiteit Leuven in accordance to The Code of Ethics of the World Medical Association (Declaration of Helsinki) [48].

### Participants

#### Experiment 1

Performance on the ‘Action Recognition Test’ and the ‘Gender Recognition Test’ was assessed in 12 males (mean age 25.1, S.D. 1.8 years) and 16 females (mean age 25.1, S.D. 3.1 years). Three out of the 28 participants were left-handed (self-reported).

#### Experiment 2

Performance on the ‘Biological Motion Recognition Test’, the ‘Emotion Recognition Test’, the ‘Control Color Test’ and the ‘Reading the Mind in the Eyes Test’ [47] was assessed for 15 males (mean age 27.1, S.D. 5.8 years) and 22 females (mean age 22.2, S.D. 4.4 years), who had not participated in Experiment 1. Due to technical problems, data on the ‘Emotion Recognition Test’ was lost for 2 male and 3 female participants.

All subjects were students at the K. U. Leuven, naive as to the purpose of the study and had no previous experience with point light displays.

### Point light displays - Motion Capturing

One male and one female actor were selected to create the point light displays (PLD). Each actor was asked to perform 5 actions, each carried out in 4 different ‘emotional states’. The **5 actions** were: (i) *walking*; (ii) *jumping* on the spot; (iii) *kicking* a ball using the right leg; (iv) *drinking* from a bottle of water, and (v) *wiping* the table. The **4 emotional states** were: (i) neutral; (ii) happy; (iii) sad, and (iv) angry. After two or more practice trials, all actions/emotions were recorded three times for each actor. One of the three recordings was selected based on the visibility of the reflective markers, leaving a grand total of 40 recorded motion scenes (‘2 actors’×‘5 actions’×‘4 emotions’).

**Figure 1:**
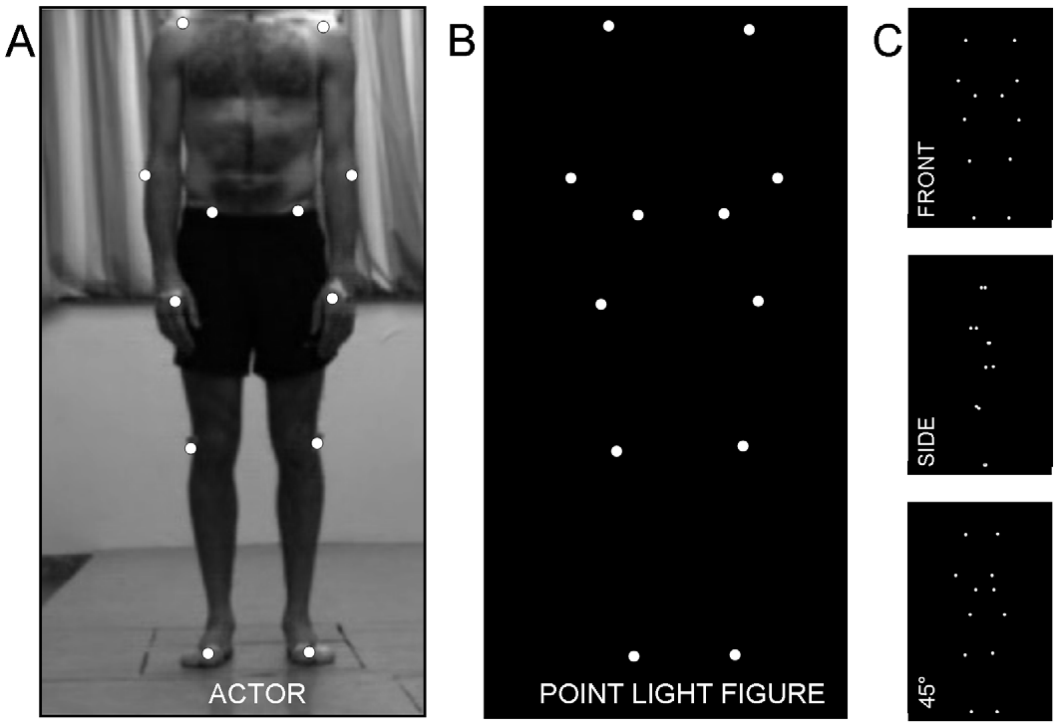
To create the point light displays, twelve reflective markers were attached to an actor's shoulders, elbows, wrists, hips, knees and ankles, and were tracked using a Vicon motion-capture-system. (**A**) An exemplary photograph of the male actor with the 12 markers attached to the body. (**B**) The corresponding point light figure. (**C**) Examples of point light figures, viewed from different perspectives i.e., the front, the side, and the 45° view.

To obtain the captured motion data, an eight-camera VICON system was used (capturing system measuring at 100 Hz, Oxford Metrics, Oxford, UK). Twelve reflective markers were attached to the joints of the ankles, the knees, the hips, the wrists, the elbows, and the shoulders of the actor (Figure 1A, 1B). After the capture session, the 2-D data from all camera units (8) were processed off line to calculate the 3-D coordinates of the markers (Vicon Motion Systems, Oxford, UK). To create the actual movie files, in house made scripts were used, created with Matlab software (MathWorks, Massachusetts, U.S.A.). For each time point, 3-D coordinates of the 12 marker dots were converted as white spheres on a black background. Frames of the captured scene were rendered as audio-video interleaved (avi) movie files at a frame rate of 20 Hz. For each recorded scene, movie files with a duration of 3 s were created from three different viewpoints (front view (0°), side view (90°), intermediate view (45°) (Figure 1C) resulting in 120 PLDs in total (‘2 actors’×‘5 actions’×‘4 emotions’, ‘3 perspectives’).

In addition, for each of the PLDs, a scrambled version was created which consisted of the same individual dots, undergoing the same local trajectories as in the normal PLDs, however with the position permutated between the 12 individual trajectories. This ‘scrambling’ resulted in 120 PLDs showing non-biological ‘scrambled’ motions.

In all the presented PLDs; the dots appeared white against a black background subtending 11×12 degrees visual angle at an approximate viewing distance of 50 cm (note that subjects were free to make small trunk movements). Each dot subtended 0.25 degrees (for example movies, see Video S1, S2 and S3).

### Experiment 1: Stimuli & Procedure

Experiment 1 consisted of 2 tests: the ‘Action Recognition Test’ and the ‘Gender Recognition Test’. Both tests were assessed in a quiet room, on the same computer monitor. Instructions were provided verbally and on the monitor at the start of each test.

Participants had to watch a series of short movies (duration of 3 s), representing PLDs of white dots against a black background. For the ***‘Action Recognition Test’***, participants were asked to indicate as fast as possible the displayed **actions** in the point light animations by pressing different buttons on a keyboard. The five response options (walking, jumping, kicking, drinking, wiping) were indicated on the respective response buttons. For the ***‘Gender Recognition Test’***, participants were asked to indicate as fast as possible the **gender** of the point light figure by pressing different buttons on a keyboard. The two response options (male, female) were indicated on the respective response buttons.

Reaction times (RT) to indicate the action or gender (from the start of the movie, until a response button was pressed), as well as accuracy rates (% correct answers) were assessed for all subjects. E-Prime software (Psychological Software Tools) was used for stimulus presentation and RT/response logging. Half of the participants started the experiment with the ‘Action Recognition Test’, the other half with the ‘Gender Recognition Test’. For each test, subjects were presented with the same set of 120 movies showing 2 genders (male and female); 5 actions (walking, jumping, kicking, drinking, wiping); and 4 emotional states (neutral, happy, sad, angry) from 3 different viewing perspectives (front view, side view and 45° view) (see Table S1). Movies were presented in a random order to each subject.

### Experiment 2: Stimuli & Procedure

Experiment 2 consisted of the ‘Biological Motion Recognition Test’, the ‘Control Color Test’, and the ‘Emotion Recognition Test’ The principal setup was identical to experiment 1.

#### ‘Biological Motion Recognition Test’

Subjects had to watch a series of PLDs (144) that either ‘moved like a person’ (‘biological motion’ PLDs (72)) or ‘moved not like a person’ (‘scrambled’ PLDs' (72)). Participants were asked to indicate as fast as possible whether the presented PLD represented “a person” or “not a person” by pressing different buttons on a keyboard. The two response options (person, no person) were indicated on the respective response buttons. The set of 72 ‘biological motion’ PLDs was obtained by a combination of 4 factors, i.e., 2 genders (male and female); 3 actions (walking, jumping, kicking); 4 emotional states (neutral, happy, sad, mad) and 3 different viewing perspectives (front view, side view and 45° view). Movies were presented in a random order to each subject.

#### ‘Control Color Test’

For this test, the same set of 144 PLDs (72 ‘biological motion’ PLDs, 72 ‘scrambled’ PLDs) was presented to the subjects. However, here participants were asked to indicate as fast as possible whether one of the moving white dots changes color to either ‘red’ or ‘green’ by pressing different buttons on a keyboard. The two response options (red, green) were indicated on the respective response buttons.

#### ‘Emotion Recognition Test’

In this test, participants were presented with a series of 144 movie trials. Each trial consisted of a ‘prime’ PLD, followed by a ‘target’ PLD. Participants were asked to indicate as fast as possible whether the presented point light figure in the ‘target’ movie performed the displayed action in a different ‘emotional state’ compared to the point light figure in the ‘prime’ movie. The emotional state of the target could either be indicated as (i) happier, (ii) sadder, (iii) angrier, or (iv) not different, from the prime. The four response options (happier, sadder, angrier, no difference) were indicated on the respective response buttons on the keyboard. Prime and target movies remained constant with respect to (i) the presented model (e.g. if the prime was male, also the target was male) and (ii) the type of action displayed (e.g., if the prime was a walking point light figure, also the target was a walking point light figure). On the other hand, the viewing perspective was always different between prime and target movies (e.g., if the prime was viewed from the front view, the target was presented either from the side view or the 45° view). The prime movie always showed a point light figure in the ‘neutral emotional state’, whereas the emotional state of the target point light figure could either be (i) neutral, (ii) happy, (iii) sad, or (iv) angry. The viewing perspective was changed between prime and target movies to increase task-difficulty and, thus, to ensure that subjects had to perceive and interpret movement kinematics rather than comparing lower-order visual properties. (e.g. the dot movement would be identical when a ‘neutral’ prime is followed by a ‘neutral’ target which could be solved by applying a visual memory strategy). The above design resulted in a grand total of 144 possible prime-target sequences, i.e., 18 prime movies (2 actors (male female)×3 actions (walking, jumping, kicking)×3 perspectives) each followed by 8 possible target movies (4 emotions (neutral, happy, sad, mad)×2 perspectives).

All participants completed the different tests in the same fixed order, starting with the ‘Biological Motion Recognition Test’, followed by the ‘Control Color Test, and finishing with the ‘Emotion Recognition Test’. This order was kept fixed such that all subjects were comparably ‘naive’ on the nature of ‘biological’ versus ‘scrambled’ PLDs in the ‘Biological Motion Recognition Test’.

Reaction times and accuracy rates were assessed for each test using E-Prime software (Psychological Software Tools).

In addition to the above tests, performance on the ***‘Reading the Mind in the Eyes Test’*** (revised version) was also administered for participants of experiment 2. This test was developed by the group of Baron Cohen et al. (2001) as a measure of adult ‘mentalizing’ and was shown to be a standardized and sensitive test to reveal subtle individual differences in facial emotion recognition. A detailed description of this test is provided elsewhere [47]. A computerized Dutch version of this test was adopted in the present study (created with Question Writer software, Central Question Ltd., Manchester, UK). In short, participants were presented with a series of 36 photographs of the eye region of the face of different actors and actresses, and were asked to indicate (by mouse clicking) which of four words best describes what the person in the photograph is thinking or feeling. Accuracy rates were assessed.

### Normative data analysis and statistics

Normative data analyses were performed to assess the overall ‘recognizability’ of the distinct features in the presented PLDs. The percentage of participants who correctly identified the displayed PLD (% correct classification score) was calculated for all PLD movies (separately for each test). In experiment 2, only PLDs of ‘walking, ‘jumping’ and ‘kicking’ were used. To make analyses and interpretations of experiment 1 and 2 comparable, only % correct classification data on these three actions were included in the analyses of experiment 1 (i.e., leaving out recorded data on the point light animations displaying the ‘drinking’ and ‘wiping’ actions). Since % correct classification scores were not normally distributed, nonparametric tests were used for the normative analyses [Shapiro-Wilk tests: all, W<.9, p<.001]. One Sample Wilcoxon Signed Rank tests were used to determine whether the group of subjects performed significantly above chance for the different tests. Additionally, non-parametric Kruskal-Wallis ANOVA tests were performed on the % correct classification scores to explore whether the recognizability of the PLDs was influenced by the (i) model's gender, (ii) the displayed action, (iii) emotion, or (iv) viewing perspective.

### Gender analyses and statistics

To explore potential differences between male and female participants in recognition performance, ANOVAs with the categorical between-group factor ‘subject's gender’ were conducted on the % correct answers and RT data, separately for all tests. Only % correct answers and RT data on the walking, ‘jumping’ and ‘kicking’ actions were included in the analyses of experiment 1 (i.e., leaving out recorded data on the point light animations displaying the ‘drinking’ and ‘wiping’ actions). Reaction times recorded from the correct trials were considered as outliers and removed from the analysis when they exceeded Q3±1.5×(Q3-Q1) with Q1 and Q3 denoting the first and third quartile over the whole set of correct trials for each subject (Electronic Statistics Textbook, 2007, StatSoft, Inc. Tulsa). Following these criteria, only a few trials were discarded from the RT analyses [‘Action Recognition Test’: 1.01%] [Gender Recognition Test’: 0.43%] [‘Biological Motion Recognition Test’: 0.88%] [‘Control Color Test’: 1.35%] [‘Emotion Recognition Test’: 0.88%]. Percentage correct answers were normally distributed for all tests [Shapiro-Wilk tests; all, W>.96, p>.1], except for the ‘Action Recognition Test’ [W = .75, p<.001] and the ‘Control Color Test’ [W = .9, p<.01]. For these variables, nonparametric Kolmogorov-Smirnov tests were adopted to compare female versus male subjects. RT data were normally distributed for all tests [W>.93, p<.001].

### Correlation analysis

Performance (% correct answers and reaction times) on the ‘Emotion Recognition Test’ was correlated to performance on the ‘Biological Motion Recognition Test’ to test whether a subject's ability to discern ‘emotional’ information from PLDs was related to a subject's ability to discriminate ‘biological’ from ‘non-biological’ motion.

In addition, correlation analysis was performed between the % correct answers of the ‘Emotion Recognition Test’ and the ‘Reading the Mind in the Eyes Test’. This was done to test the relationship between the ability to recognize emotions from *bodily* PLD kinematics as compared to photographs statically showing the eye region. As a control, correlations with the aforementioned tests and the ‘Color test’ were performed.

All statistics were calculated with Statistica 9.0 (StatSoft. Inc. Tulsa, USA).

## Results

### Normative Data analysis

For all the presented PLDs (72), Table S1 reports the % correct classification scores, separately for all tests.

#### ‘Action Recognition Test’

Participants were able to identify each of the actions (walking, jumping, and kicking) reliably and far above chance level [% correct above chance for ‘walking’: 78.9%; ‘jumping’: 77.5%; and ‘kicking’: 77.3%] as confirmed by one Sample Wilcoxon Signed Rank tests [all, Z(23)>4.2; p<.001]. Kruskal-Wallis ANOVA analyses only revealed a significant main effect of ‘Emotion’ [H(3,72) = 13.5, p = .004], which indicated that slightly more subjects recognized the displayed action from the ‘angry’ point light figure [99.6%, SEM 0.3], than from the ‘happy’ [96.2%, SEM 0.8] point light figure. The effects of ‘Model's gender’ [H(1,72) = .23, p = .63], ‘Action’ [H(2,72) = 3.1, p = .21], and ‘Perspective’ [H(2,72) = 4.2, p = .12] were not significant, indicating that action recognition was not influenced by these factors.

#### ‘Gender Recognition Test’

Participants were able to identify the gender of the point light figure only slightly above chance level [7.5%] [Z(71) = 3.0; p = .003].

Kruskal-Wallis ANOVA analyses revealed no significant effects [all, H<5.4, p>.05], indicating that gender identification was not influenced by the (i) model's gender, (ii) action, (iii) emotion, or (iv) viewing perspective.

#### ‘Biological Motion Recognition Test’

Participants were able to identify the ‘biological motion’ versus ‘scrambled’ PLDs reliably and above chance level [% correct above chance for ‘biological’: 42.6%; and ‘scrambled’: 36.9] [both, Z(71)>7.3; p<.001].

Kruskal-Wallis ANOVA analyses were performed only for the % correct classification scores of the ‘biological motion’ PLD (72) (not for the ‘scrambled’ PLD). A significant main effect for the factor ‘Emotion’ [H(3,72) = 9.2, p = .03] indicated that slightly fewer subjects recognized ‘biological motion’ from the ‘sad’ PL figure [87.7%, SEM 2.1], than from the ‘angry’ PL figure [93.4%, SEM 2.2]. Additionally, a main effect of ‘Perspective’ [H(2,72) = 21.9 p<.001] indicated that ‘biological motion’ recognition was the most difficult from the side view [87.5%, SEM 1.9], intermediate for the 45° view [92.8%, SEM 1.5], and the least difficult from the front view [97.6%, SEM 0.6]. The effects of ‘Model's gender’ [H(1,72) = 3.3, p = .07] and ‘Action’ [H(2,72) = 4.5, p = .12] were not significant.

#### ‘Emotion Recognition Test’

Participants were able to identify each of the emotions (neutral, happy, sad, angry) reliably and above chance level [% correct above chance for ‘neutral’: 54.3%; ‘happy’: 44.2%; ‘sad’: 45.8%; and ‘angry’: 58.6%] [all, Z(35)>4.9; p<.001].

Kruskal-Wallis ANOVA analyses revealed no significant effects [all, H<6.9, p>.05], indicating that emotion recognition was not influenced by the (i) model's gender, (ii) action, (iii) emotion, or (iv) viewing perspective.

### Female versus male differences in recognizing bodily kinematics

Potential gender differences in recognition performance were assessed for all tests.

#### ‘Action Recognition Test’ (Experiment 1)

**Figure 2:**
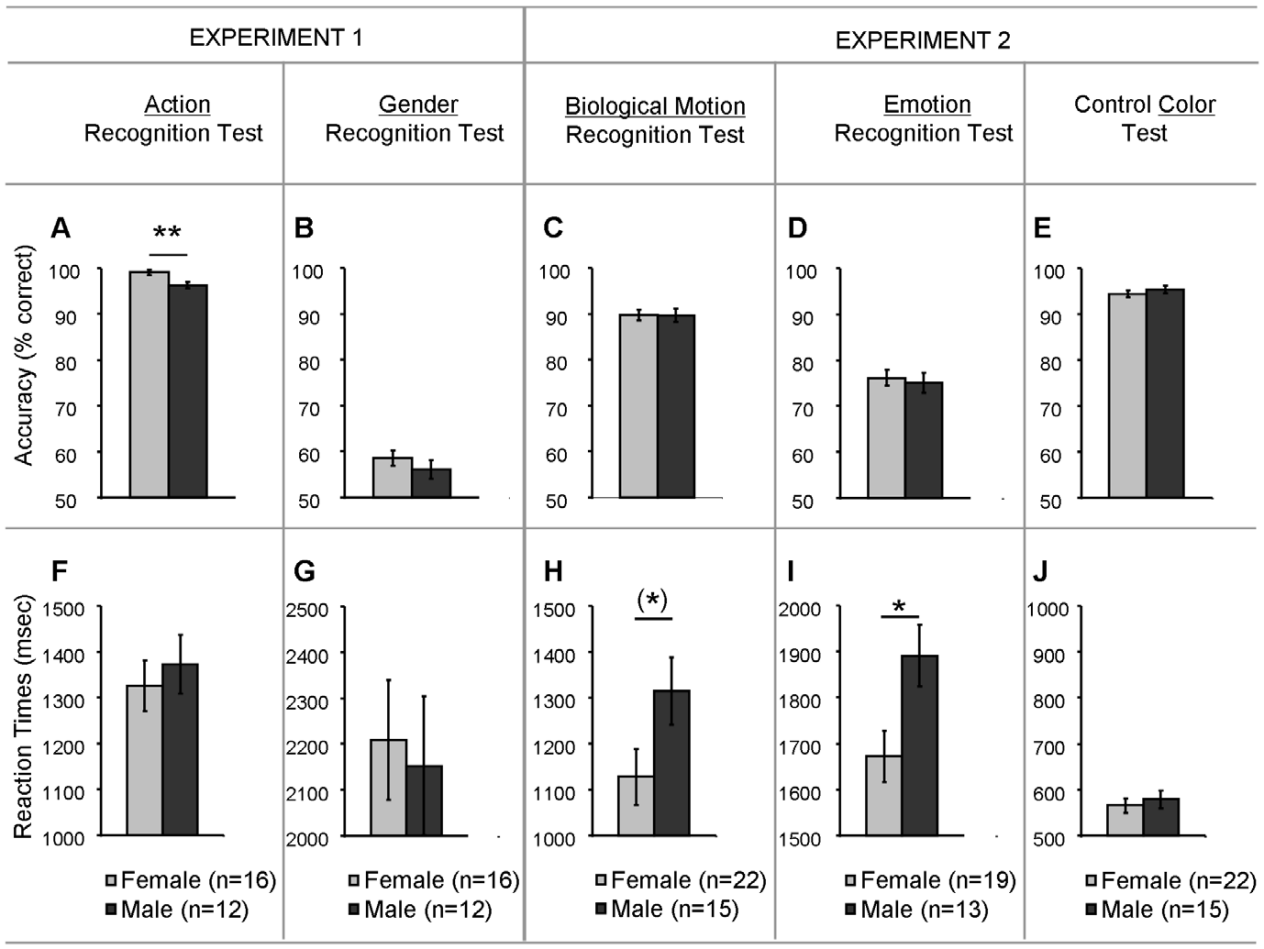
Gender differences in test performance. Accuracy (% correct scores) (A–E) and Reaction times (F–J) are displayed as a function of participant group (male, female) separately for each test of experiment 1 [Action Recognition Test (A, F); Gender Recognition Test (B, G)] and experiment 2 [Biological motion Recognition Test (C, H); Emotion Recognition Test (D, I); Control Color Test (E, J)]. Vertical lines denote ±standard error. [(*) p = .05; * p<.05; ** p<.01].

A Kolmogorov-Smirnov test on the % correct answers revealed a significant effect of gender [p<.05], indicating better action recognition for the female compared to the male participants (Figure 2A). Reaction times were comparable for both genders [F(1, 26) = .32, p = .58] (Figure 2F).

#### ‘Gender Recognition Test’ (Experiment 1)

Gender recognition from the PLDs was shown to be comparable for female and male participants, both in terms of % correct answers [F(1, 26) = .96, p = .34] (Figure 2B) and reaction times [F(1, 26) = .07, p = .78] (Figure 2G).

#### ‘Biological Motion Recognition Test’ (Experiment 2)

As a group, females were shown to be tentatively faster compared to males in recognizing a ‘biological actor’ or ‘person’ from the PLDs. This was revealed by a significant effect of gender from the one-way ANOVA analysis on the RT data [F(1, 35) = 3.8, p = .05] (Figure 2H). Percentage correct answers were shown to be comparable for both groups [F(1, 26) = .007, p = .93] (Figure 2C).

#### ‘Emotion Recognition Test’ (Experiment 2)

Similar to findings from the ‘Biological Motion Recognition Test’, females were shown to be significantly faster compared to males in recognizing the displayed emotions from the presented PLDs. This was revealed by a significant effect of gender from the one-way ANOVA analysis on the RT data [F(1, 30) = 6.2, p = .017] (Figure 2I). Percentage correct answers were shown to be comparable for both groups [F(1, 30) = .14, p = .72] (Figure 2D). We additionally explored the potential interaction between ‘Emotion’ and ‘Subjects' gender’ with a repeated measures ANOVA on the % correct answers and RT data (with the within factor ‘Emotion’ and the between factor ‘Subjects' gender’). However, no significant interaction effects were revealed [Acc: F(3,90) = .09, p = .96] [RT: F(3,90) = .4, p = .76], indicating that gender effects were similar for all types of emotion.

#### ‘Control Color Test’ (Experiment 2)

Reaction times to indicate the color change of the dot, as well as % correct answers were shown to be comparable for both genders [RT: F(1, 35) = .29, p = .59] (Figure 2J) [Acc: p>.1] (Figure 2E).

#### ‘Reading the Mind in the Eyes Test’ (Experiment 2)

One-way ANOVA analysis on the % correct answers revealed a significant effect of gender [F(1, 35) = 6.3, p = .016], replicating previous findings of a female superiority on the *‘Reading the Mind in the Eyes Test’* [47], [49].

### Correlated performance

**Figure 3:**
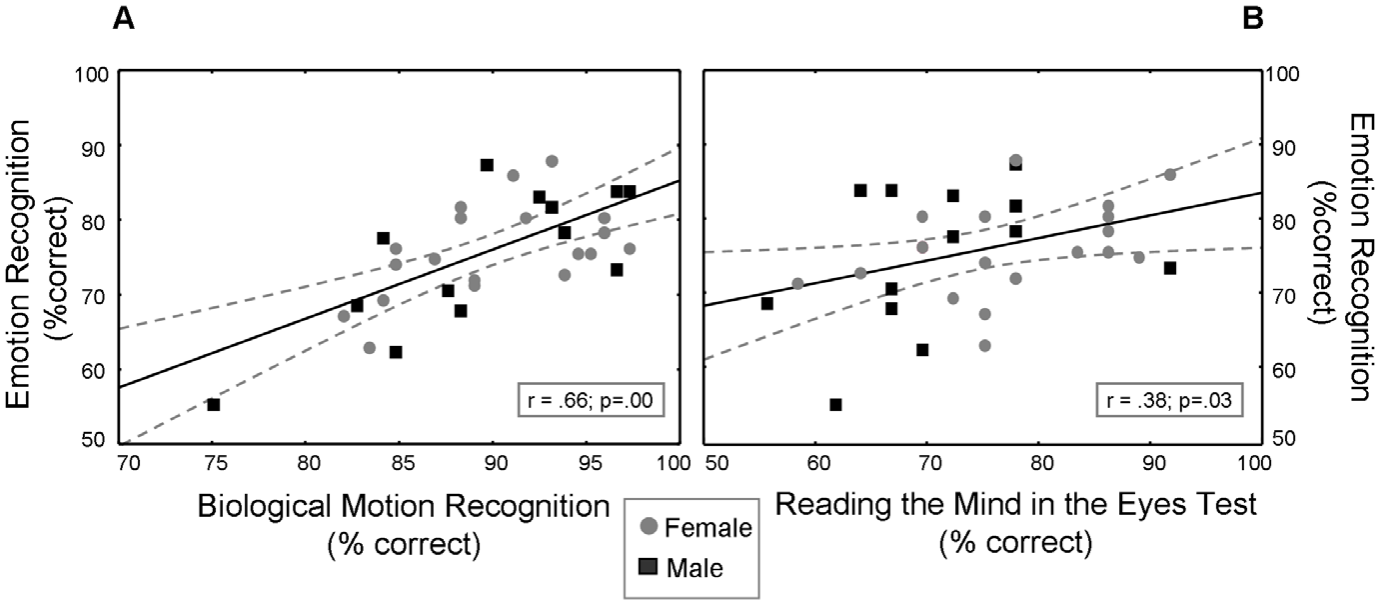
Performance on the ‘Emotion Recognition Test’ correlates with performance on the ‘Biological Motion Recognition Test’ and the ‘Reading the Mind in the Eyes Test’. Figure 3 shows linear fits for correlated performance (% correct answers) on the ‘Emotion Recognition Test’ and respectively the ‘Biological Motion Recognition Test’ (A) and the ‘Reading the Mind in the Eyes Test’ (B) of experiment 2. Dotted lines denote 0.95 confidence intervals.

Performance on the ‘Emotion Recognition Test’ was shown to correlate significantly with performance on the ‘Biological Motion Recognition Test’, both in terms of accuracy [r = .66; p<.001] (Figure 3A) and reaction times [r = .56; p<.001] (not shown in figure). These correlations indicate that a subject's ability to discern emotional information from PLDs was related to the subject's ability to basically discriminate ‘biological’ from ‘non-biological’ motion.

A significant correlation was also obtained between performance on the ‘Emotion Recognition Test’ and the ‘Reading the Mind in the Eyes’ Test [r = .38; p = .03] (Figure 3B), i.e., indicating that subjects scoring high on the ‘Reading the Mind in the Eyes Test’ were also good at recognizing *bodily* emotions from PLDs. No correlations were found between performance on the ‘Control Color Test’ and all other tests [all, r<.2; p>.1].

## Discussion

The aim of the present study was to provide an objective quantification of potential gender differences in a set of socio-cognitive tasks involving the recognition of distinct features from point light displays (PLDs). Females were shown to perform better than male participants on PLD perception tasks involving bodily action recognition (experiment 1) and bodily emotion recognition (experiment 2). Interestingly, gender differences even pertained to tasks involving the basic recognition of ‘biological’ from ‘non-biological’ PLD motion (experiment 2). Moreover, previous findings of a female superiority on the ‘Reading the Mind in the Eyes test’ were replicated in the present study. No gender differences were revealed for the control test (indicate color change in one of the moving dots) and for recognizing the gender of the PLD figure. Interestingly, accuracy scores on the ‘Reading the Mind in the Eyes test’ were shown to predict the subjects' accuracy in recognizing emotions from *bodily* kinematics depicted by PLDs.

In experiment 1, gender differences were revealed for the ‘Action Recognition Test’ in terms of accuracy scores, such that females produced more correct answers compared to males, with comparable or even tentatively faster reaction times. The finding that performance was comparable for males and females on the ‘Gender Recognition Test’ suggests that the observed gender effect in action recognition is not related to general gender differences in reaction times or response selection abilities (i.e., selecting the correct finger to the correct response button). Overall, the lack of differences between male and female participants on the ‘Gender Recognition Test’ accords to some previous studies also showing no gender differences in gender recognition from PLDs [50], [51]. Moreover, overall gender recognition accuracy was only slightly above chance, potentially because the actors shown in the PLDs provided only subtle gender specific cues. A recent study exploring gaze patterns during gender recognition from PLDs suggested that the primary cues to gender are found in the shoulder and pelvis or ‘hip-shoulder ratio’ of a point light walker [52]. Results from our and previous studies suggest that the processing of these gender-specific cues (i.e., relating to body structure, and in particular to the relative width of shoulders and hips) may be comparable between genders.

In experiment 2, male participants appeared to adopt a more ‘time consuming’ strategy to accomplish the task compared to females both in the ‘Biological Motion’, and ‘Emotion Recognition Tests’, (as revealed by higher reaction times for males compared to females). Also here, this male-female difference seems task-specific, since reaction times on the ‘Control Color Test’ were comparable for the same male and female participants. It should be noted that the control task was relatively easy such that, theoretically, there might have been a ceiling effect in reaction times. However, even this relatively simple choice reaction time task is sufficient to control for potential gender differences in generating fast motor responses. Moreover, in light of the data from experiment 1 that revealed *no* gender differences on the (rather demanding) Gender recognition test, it seems unlikely that the gender effects observed in experiment 2 are solely driven by differences in response time or response selection abilities.

Overall, the finding of sex differences on bodily emotion recognition from point light animations is consistent with previous studies reporting gender differences in emotion recognition from facial expressions [6], [7], [53], [54]. A standardized and sensitive test that has been developed in this context is the ‘Reading the Mind in the Eyes Test’, in which subjects are presented with a series of photographs of the eye region of the face of different persons, and are asked to indicate which of four words best describes what the person in the photograph is thinking or feeling [47]. In the present study we were able to replicate previous findings of a female superiority in facial emotion recognition [47], [49]. Moreover, performance on this ‘facial’ emotion recognition test was shown to correlate significantly with performance on our newly developed ‘bodily’ emotion recognition test. Together, these findings indicate that gender differences in visually recognizing emotions are relatively generalized across *facial* and *bodily* emotion perception (i.e., irrespective of whether the task involves the ‘reading’ of fine movements in facial muscles, or the ‘embodiment’ of whole body emotional states). In addition, results from our Action and Biological Motion Recognition Tests provide indications that these gender differences are not restricted to the emotional domain, but are also manifest in tasks involving more ‘general’ biological motion processing.

Although substantially different in methodological approach, some recent studies explored differences between males and females in understanding actions and intentions from a visual event arrangement task. In this task, participants have to organize a set of cards depicting an event as a series of snapshots in a comic-strip fashion (i.e., requiring the understanding of the intentions and dispositions of the characters involved in the events) [55], [56]. In contrast to our results, these studies found **no** difference in action and intention understanding between female and male participants. However, the neural mechanisms of action and intention understanding from such reconstruction tasks (using *static* frames) might substantially differ from our *dynamic* PLD perception paradigms.

Our findings are consistent with previous studies reporting gender differences at the neurophysiological level for areas that are part of the action observation system and that respond to biological motion perception from PLDs [31]–[35]. In this respect, the presently reported differences between males and females in PLD recognition tasks may provide first indications that these neurophysiological differences are also relevant and quantifiable at the behavioral level. Interestingly, abundant reports exist on gender-related differences on empathy scores as indexed by standard questionnaires [1], [1]–[4], [57]–[60], [60,61] and also MNS activity has been shown to correlate to empathy indices [35], [62], [63]. In this respect, although rather speculative, it can be envisaged that inter-individual or gender-dependent differences in emotional processing may - at least to some degree – be related to more basic differences in processing biological motion per se. Results from our correlation analyses speak in favor of this notion by showing a tight relationship between a subject's ability to discern subtle emotional cues from PLDs and the subject's ability to basically discriminate biological from non-biological motion. Similar findings were revealed from a previous study examining a link between explicit detection of human gait from PLDs and emotion recognition [64]. Here however, only significant correlations with gait detection performance were observed for detecting ‘angry’ emotional states but not for ‘happiness’ detection.

Contrary to the notion of gender differences in the ‘biological motion processing network’, a recent magneto-encephalography study showed that gender-dependent differences in acquiring social information are related to differences in regions of the perceptual decision making network, namely, when the presence of social interactions had to be judged based on animated motions of geometric shapes [65]. Overall, it is not surprising that these non-biological stimuli produced effects outside the MNS, namely within the left prefrontal cortex, a region implicated in perceptual decision making. In addition, results indicated that females judged the presence of social interaction more rapidly than males who seemed to require more sensory evidence for social decisions [65]. Despite the apparent difference in adopted stimuli between this and our study, it should be interesting for future research to address whether the presently reported gender differences on PLD perception tasks are related to differences in the biological motion processing network, the perceptual decision making network, or in both systems. In general, such investigations may also shed light on gender-related vulnerabilities to neuropsychiatric disorders that are characterized by social cognition problems, such as autism spectrum disorders (ASD). Impairments in PLD biological motion perception have been reported in ASD [66]–[69] and males are known to be more commonly affected by the disorder than females with a ratio of about 4∶1 [70]. In this respect, it should be interesting to explore whether the normal gender difference in response to our point light action, biological motion and emotion recognition tasks is even more pronounced in ASD.

## Supporting Information

Table S1 **Reported values refer to the percentage of participants who correctly identified each feature of the presented point light displays (N = 72) i.e., (i) the ‘type of action’ in the Action Recognition test, (ii) the ‘model's gender’ in the Gender Recognition test, (iii) a ‘person’ in the Biological Motion Recognition test, and (iv) the ‘type of emotion’ in the Emotion Recognition test.** [*Sample of 28 participants; **Sample of 37 participants; ***Sample of 32 participants].
(PDF)

Video S1 **Exemplary movie of a point light display consisting of 12 moving white dots against a black background.** The point light display shows the female actor walking on the spot (neutral emotional state, side view).
(WMV)

Video S2 **Scrambled version of the point light display showed in Video S1.** It consists of the same individual dots, undergoing the same local trajectories as in the normal point light display, however with the position permutated between the 12 individual trajectories.
(WMV)

Video S3 **Exemplary movie of a ‘sad’ point light figure (female actor walking on the spot, front view).**
(WMV)
