# Supplementary material for: Action and Emotion Recognition from Point Light Displays: An Investigation of Gender Differences
Source: PLoS One. 2011 Jun 9;6(6):e20989. doi: 10.1371/journal.pone.0020989 (PMC3111458; doi:10.1371/journal.pone.0020989)
Supplement: Table S1 — Reported values refer to the percentage of participants who correctly identified each feature of the presented point light displays (N = 72) i.e., (i) the ‘type of action’ in the Action Recognition test, (ii) the ‘model's gender’ in the Gender Recognition test, (iii) a ‘person’ in the Biological Motion Recognition test, and (iv) the ‘type of emotion’ in the Emotion Recognition test. [*Sample of 28 participants; **Sample of 37 participants; ***Sample of 32 participants]. (PDF) [file pone.0020989.s001.pdf]

SUPPORTING INFORMATION

Table S1

Table S1

Normative Data for all the presented Point light displays (PLD)

PLD	Action	Emotion	Model	Perspective	Correct Classification (%) *Action Recognition Test	*Gender Recognition Test	**Biological Motion Recognition Test	***Emotion Recognition Test
1	Walk	Neutral	F	Front	100	68	100	63
2	Walk	Neutral	F	Medium	100	54	97	88
3	Walk	Neutral	F	Side	100	79	97	67
4	Walk	Sad	F	Front	100	25	97	98
5	Walk	Sad	F	Medium	100	32	81	95
6	Walk	Sad	F	Side	96	43	92	83
7	Walk	Happy	F	Front	96	75	97	75
8	Walk	Happy	F	Medium	100	57	97	53
9	Walk	Happy	F	Side	96	64	97	56
10	Walk	Angry	F	Front	100	29	97	70
11	Walk	Angry	F	Medium	100	57	97	67
12	Walk	Angry	F	Side	100	61	95	81
13	Jump	Neutral	F	Front	89	79	95	86
14	Jump	Neutral	F	Medium	100	82	100	84
15	Jump	Neutral	F	Side	96	71	73	84
16	Jump	Happy	F	Front	93	57	100	38
17	Jump	Happy	F	Medium	93	64	84	23
18	Jump	Happy	F	Side	100	71	73	9
19	Jump	Angry	F	Front	100	46	100	94
20	Jump	Angry	F	Medium	100	54	97	91
21	Jump	Angry	F	Side	100	75	92	88
22	Jump	Sad	F	Front	100	36	100	89
23	Jump	Sad	F	Medium	100	71	95	88
24	Jump	Sad	F	Side	96	57	84	84
25	Kick	Neutral	F	Front	100	82	100	69
26	Kick	Neutral	F	Medium	96	64	100	83
27	Kick	Neutral	F	Side	100	82	92	94
28	Kick	Sad	F	Front	96	43	97	84
29	Kick	Sad	F	Medium	100	29	97	72
30	Kick	Sad	F	Side	100	50	89	63
31	Kick	Happy	F	Front	93	29	97	75
32	Kick	Happy	F	Medium	96	25	97	61
33	Kick	Happy	F	Side	93	25	92	67
34	Kick	Angry	F	Front	100	36	97	97
35	Kick	Angry	F	Medium	100	25	100	95

36	Kick	Angry	F	Side	100	29	100	91
37	Walk	Sad	M	Front	96	86	100	72
38	Walk	Sad	M	Medium	100	75	97	84
39	Walk	Sad	M	Side	100	57	95	83
40	Walk	Neutral	M	Front	100	82	86	97
41	Walk	Neutral	M	Medium	100	71	84	98
42	Walk	Neutral	M	Side	100	43	95	97
43	Walk	Happy	M	Front	96	86	97	25
44	Walk	Happy	M	Medium	96	82	95	30
45	Walk	Happy	M	Side	100	71	97	28
46	Walk	Angry	M	Front	96	86	97	78
47	Walk	Angry	M	Medium	100	86	100	88
48	Walk	Angry	M	Side	100	64	86	97
49	Jump	Neutral	M	Front	96	57	97	78
50	Jump	Neutral	M	Medium	100	32	78	80
51	Jump	Neutral	M	Side	96	32	84	78
52	Jump	Sad	M	Front	96	71	95	72
53	Jump	Sad	M	Medium	96	39	81	50
54	Jump	Sad	M	Side	100	46	76	36
55	Jump	Angry	M	Front	100	79	97	89
56	Jump	Angry	M	Medium	100	61	92	92
57	Jump	Angry	M	Side	96	50	86	92
58	Jump	Happy	M	Front	93	71	100	69
59	Jump	Happy	M	Medium	100	46	86	77
60	Jump	Happy	M	Side	96	36	78	72
61	Kick	Sad	M	Front	82	82	97	67
62	Kick	Sad	M	Medium	96	68	95	80
63	Kick	Sad	M	Side	100	25	89	88
64	Kick	Angry	M	Front	100	79	97	88
65	Kick	Angry	M	Medium	100	64	81	89
66	Kick	Angry	M	Side	100	57	73	83
67	Kick	Neutral	M	Front	96	79	100	80
68	Kick	Neutral	M	Medium	96	57	95	80
69	Kick	Neutral	M	Side	100	21	97	70
70	Kick	Happy	M	Front	100	68	100	78
71	Kick	Happy	M	Medium	100	36	68	89
72	Kick	Happy	M	Side	89	39	100	95

Reported values refer to the percentage of participants who correctly identified each feature of the presented point light displays (72) i.e., (i) the 'type of action' in the Action Recognition test, (ii) the 'model's gender' in the Gender Recognition test, (iii) a 'person' in the Biological Motion Recognition test, and (iv) the 'type of emotion' in the Emotion Recognition test.

[*Sample of 28 participants; **Sample of 37 participants; ***Sample of 32 participants]
